# The role of volunteer older adults in the exercise programme of the Comprehensive Resilience‐Building Psychosocial Intervention (CREST) for people with dementia

**DOI:** 10.1002/alz.087402

**Published:** 2025-01-09

**Authors:** Dympna Casey, Siobhán Smyth, Priscilla Doyle, Niamh Gallagher, Barbara Whelan

**Affiliations:** ^1^ University of Galway, Galway Ireland

## Abstract

**Background:**

The CREST intervention included three components one of which was an eight‐week exercise programme. The aim of the programme was to increase the exercise capacity of people with mild to moderate dementia and facilitate social engagement with volunteer older adults from the community supporting a person with dementia, raising awareness and understanding of dementia. We explore the experience of volunteer older adults’ participation in the exercise component of the CREST intervention following a five‐hour training on dementia awareness and the CREST exercise programme.

**Method:**

Qualitative semi‐structured focus groups and individual interviews were conducted with the older adults, people with dementia, their carers and the facilitators of the exercise programme. Data were analysed thematically. Older adults also completed the Dementia Attitude Scale (DAS) and the Dementia Knowledge Twenty (DK‐20) questionnaire pre‐ and post‐intervention.

**Result:**

Overall, the older adults (n = 9), people with dementia (n = 9), their carers (n = 9) and the programme facilitators (n = 2) were positive about the inclusion of the older adults in the exercise programme. Most commented on the role of the older adults in encouraging and supporting the people with dementia with the exercises and the social aspect. Two of the people with dementia highlighted difficulties in personality but they found a way to work together as the weeks progressed. While the majority of older adults felt that the training they received prior to the intervention prepared them sufficiently to support the person with dementia, there were only slight improvements in attitudes and knowledge scores about dementia based on the DAS (baseline M = 105; SD = 9.68; post‐intervention M = 109; SD = 8.80) and DK‐20 (baseline M = 11.3; SD = 4.74; post‐intervention M = 14.2; SD = 2.59).

**Conclusion:**

The volunteer older adults actively contributed to the exercise program, providing encouragement and support to the people with dementia, and enhancing the social dynamics of the sessions. There were modest improvements in the attitudes and knowledge scores about dementia among the volunteer older adults, suggesting a potential positive impact of the intervention on their awareness and understanding.

